# Acidic extracellular pH of tumors induces octamer-binding transcription factor 4 expression in murine fibroblasts *in vitro* and *in vivo*

**DOI:** 10.1038/srep27803

**Published:** 2016-06-15

**Authors:** Avik Som, Sharon Bloch, Joseph E. Ippolito, Samuel Achilefu

**Affiliations:** 1 Department of Radiology, Washington University in St. Louis School of Medicine, St. Louis, Missouri, 63110, USA; 2 Department of Biomedical Engineering, Washington University in St. Louis School of Medicine, St. Louis, Missouri, 63110, USA; 3 Department of Genetics, Washington University in St. Louis School of Medicine, St. Louis, Missouri, 63110, USA; 4 Department of Biochemistry and Molecular Biophysics, Washington University in St. Louis School of Medicine, St. Louis, Missouri, 63110, USA

## Abstract

Octamer-binding transcription factor 4 (OCT-4) is an important marker of cellular de-differentiation that can be induced by environmental stressors, such as acidity. Here we demonstrate that chronic acidic stress in solid tumors induced OCT-4 expression in fibroblasts and other stromal cells in four tumor models. The results have implications for how tumors utilize pH modulation to recruit associated stromal cells, induce partial reprogramming of tumor-associated stromal cells, and respond to therapy.

Tumor-stromal interactions play important roles in tumor growth and metastasis with direct impact on extracellular matrix enzymes, cytokines, matrix polymers such as collagen and hyaluronic acid derivatives, hypoxia, and pH^1^,^2^,^3^,^4^,^5^. Previous studies have shown that stromal cells, such as cancer-associated fibroblasts and perivascular cells, participate in tumor metastasis and growth^6^,^7^. A vexing question is how tumors recruit and alter stromal cells, including fibroblasts, from normal tissue during cancer growth and metastasis^8^,^9^,^10^.

The cancer microenvironment replicates in many ways the characteristics of normal stem cell environments^11^,^12^,^13^. Several factors, such as hypoxia and the acidic environment found in the cancer stem cell niche, are also stressors on cell metabolism and division^11^,^14^. Literature suggests that these same stressors can induce at least a partial reprogramming of fibroblast cells *in vitro*, resulting in the expression of OCT-4 in culture under conditions of metabolic stress^15^. Both *in vivo* and *in vitro* studies have shown that OCT-4 mediates cellular development. Given that the *in vivo* microenvironment of many solid tumors is weakly acidic (pH 6.5)^16^,^17^,^18^,^19^, we hypothesized that acid-mediated chronic mild stress would induce a partial reprogramming of stromal cells *in vivo*, as indicated by increased OCT-4 expression. Our results show that *in vitro* replication of the chronic mildly acidic solid tumor environment induced OCT-4 expression in murine fibroblasts. We also demonstrated that OCT-4 expression increased in fibroblasts and other supporting stromal cells within tumor xenografts. This expression of OCT-4 may explain some of the stem cell-like phenotypes of stromal cells within the tumor microenvironment.

## Results

### Exposure of cells to acidic environment induces OCT-4 expression *in vitro*

**Figure 1:**
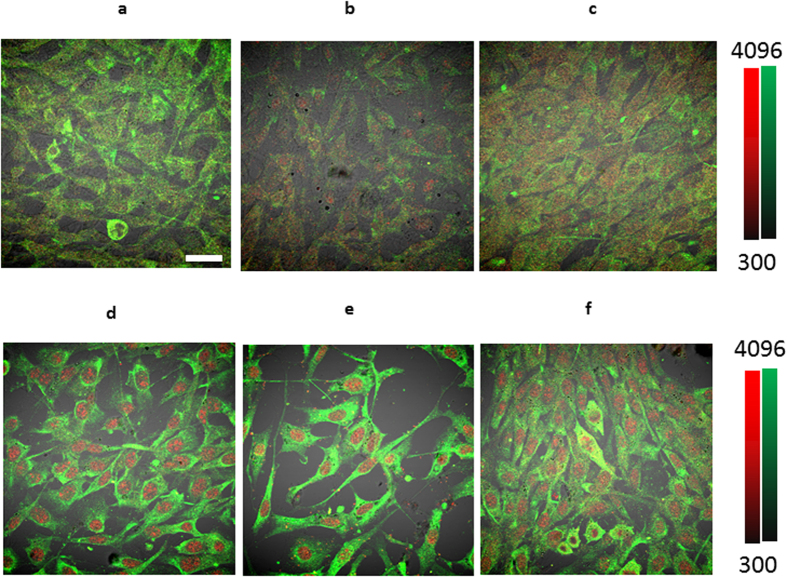
Simulation of tumor-associated pH environment *in vitro*. Confocal imaging of rabbit polyclonal anti-OCT4 (red) staining and anti-vimentin (green) in 3T3 cells under various conditions. Fibroblasts (**a**) incubated in pH 7.4 media, (**b**) co-cultured with MDA-MB-231 cells in pH 7.4 media, (**c**) co-cultured with BXPC-3 in pH 7.4 media, (**d**) incubated in pH 6.5 media, (**e**) co-cultured with MDA-MB-231 cells in pH 6.5 media, (**f**) co-cultured with BXPC-3 breast cancer cells in pH 6.5 media. All the cells were incubated for 7 days with no changes in media. Magnification is 20x. Scale bar is 100 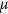
m.

We used four tumor cell lines for this study: (1) the human breast cancer cell line MDA-MB-231 is OCT-4 positive and expresses the OCT-4 inducing leukemia inhibitory factor, LiF^20^; (2) the murine mammary carcinoma EMT-6 undergoes epithelial-mesenchymal transition; (3) the human pancreatic adenocarcinoma BxPC-3 has not, to our knowledge, been shown to express OCT-4 ; and (4) the murine breast cancer model 4T1luc is an aggressive tumor model with significant drug resistance. We first determined if the tumor cells could induce OCT-4 expression in nontumor cells under normal physiologic pH. Using BxPc-3 and MDA-MB-231 cells, we cultured GFP transfected 3T3 fibroblast cells (non-tumor cells) in pH 7.4 media for 7 days in the presence or absence of tumor cells. In the absence of cancer cells, OCT-4 expression in the 3T3 cells did not increase over time (Fig. 1a). In contrast, co-culture of these fibroblasts with tumor cells at pH 7.4 resulted in increased OCT-4 expression (Fig. 1b,c). The enhanced nuclear localization of OCT-4 indicates that tumor cells can facilitate OCT-4 expression in surrounding healthy tissue under normal physiological pH.

**Figure 2:**
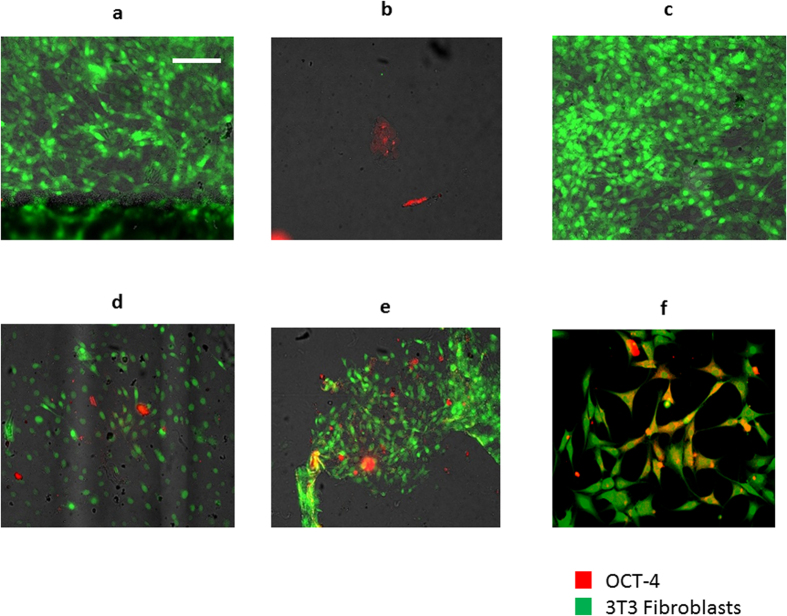
Time course of OCT-4 expression. (**a**) OCT-4 expression of GFP^+^ fibroblasts cultured for 7 days in normal pH 7.4 media. (**b**) OCT-4 expression MDA-MB-231 cells, positive control. (**c**) OCT-4 expression of GFP^+^ fibroblasts cultured for 6 days in normal pH 7.4 media and 1 day in pH 6.5 media. (**d**) OCT-4 expression of GFP^+^ fibroblasts cultured for 4 days in normal pH 7.4 media and 3 day in pH 6.5 media. (**e**) OCT-4 expression of GFP^+^ fibroblasts cultured for 2 days in normal pH 7.4 media and 5 day in pH 6.5 media. (**f**) OCT-4 expression of GFP^+^ fibroblasts cultured for 7 days in pH 6.5 media. Red represents OCT-4^+^ cells, and green represents GFP + fibroblasts. Scale bar represents 100 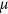
m.

A previous study showed that cells cultured in a highly acidic environment (pH 5) showed increased OCT-4 expression^15^. Because the extracellular pH of solid tumors rarely falls below pH 6, we investigated whether OCT-4 expression could be induced in cells cultured in a more physiologically relevant, mildly acidic medium (pH 6.5), similar to the extracellular pH of solid tumors. GFP transfected 3T3 fibroblast cells were cultured in acidic (pH 6.5) media for 7 days in the presence or absence of tumor cells. A high level and intense nuclear localization of OCT-4 expression was observed in both the presence and absence of tumor cells (Fig. 1d–f), suggesting the possibility of cell reprogramming under these conditions. Co-staining with vimentin shows strong cytoplasmic vimentin positivity confirming the fibroblast origin of the cells (Fig. 1). The percentage of OCT-4 positive cells sequentially increased over time (Fig. 2), indicating that chronic exposure of these cells to acidic environments such as those found in some solid tumors, provides a potential pathway to induce cellular stress to surrounding healthy cells. In addition, there is an inverse correlation of OCT-4 expression with acidity, OCT-4 intensity increasing as pH decreases from 7.4 to 6.5 (Supplementary Figure 2). Very few BxPC-3 and MDA-MB-231 (GFP-negative) cells were observed because these cells do not remain adherent during staining. Similar results were obtained when cells were stained using a second OCT-4 antibody (Supplementary Figure 1). Incubation with non-tumor cell lines, such as primary mammary epithelial cells (PMEC), under acidic conditions showed OCT-4 expression in both cell lines, while no OCT-4 expression was seen under non-acidic conditions. (Supplementary Figure 3).

### *Ex vivo* expression of OCT-4 in tumor tissue

**Figure 3:**
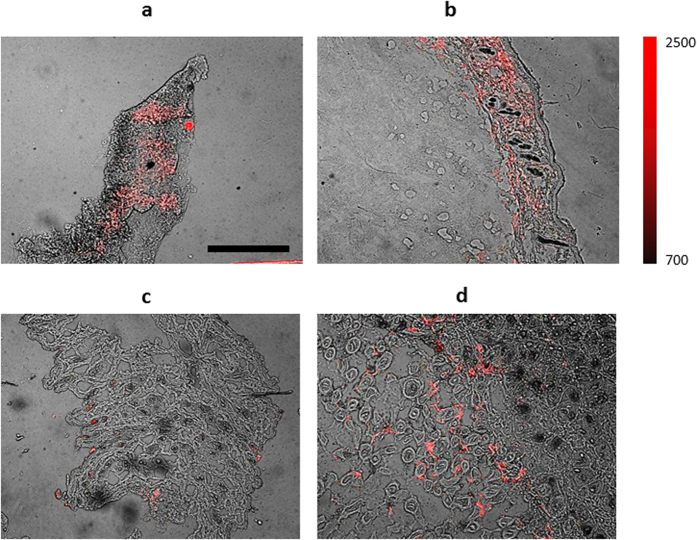
Murine Monoclonal anti-OCT-4 staining of tumor and stroma. Oct-4 expression (red) in (**a**) 4T1luc tumor; (**b**) EMT-6; (**c**) 4T1 luc tumor margin; **(d**) BxPC-3 tumor. Scale bar is 500 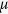
m.

We next investigated OCT-4 expression in xenografts from three tumor models: 4T1luc, EMT-6, and BxPC-3. Cardiac muscle was used as an *in vivo* negative control due to the low propensity of that tissue to stem cell activity. Expectedly, we did not detect OCT-4 expression in heart tissue (Supplementary Figure 6). Tumors were 0.5 cm to 1.5 cm in size on excision. The average pH level in 4T1 tumor models was 6.8+/− 0.1 pH units as measured by an invasive 5 mm probe. This is consistent with previous studies, which reported acidity in BxPC-3 and EMT-6 tumor models and the importance of the pH for chemotherapy resistance^19^. In contrast, OCT-4 expression was detected in all three tumor tissues (Fig. 3) and was localized to certain cells in and around the tissue section. The entirety of the section was considered to be tumor. These results suggest that OCT-4 expression spans multiple human and murine tumor types. OCT-4 expression, however, seems to be sparse, with only a few tissue areas showing positive staining.

### Identification of fibroblasts expressing OCT-4 in tumor tissue

**Figure 4:**
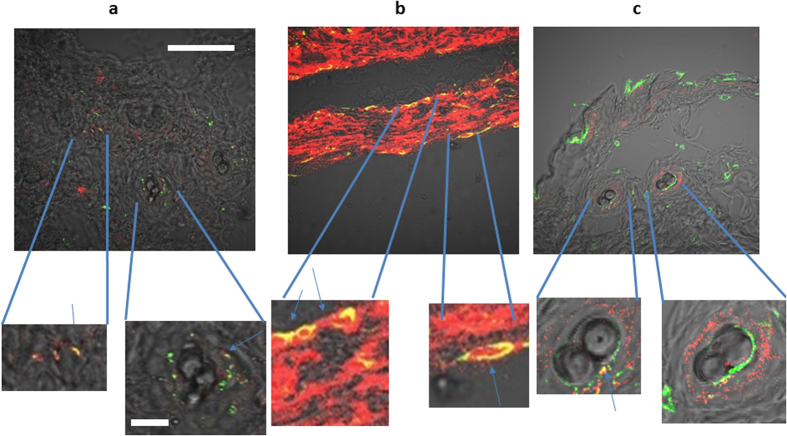
Co-staining for OCT-4 (red) & Vimentin (green) in tumor tissues. 10x bright field images with crop-outs are subsets of the original 10X images. (**a**) Co-staining of BxPC-3 tumor tissue (**b**) Staining of MDA-MB-231 tumor tissue (**c**) Staining of EMT-6 tumor Yellow indicates areas of co-localization. Scale bar represents 500 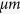 for the low magnification image, and 100 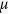
m for magnified regions.

Fibroblasts are an important and common cell type in cancer stromal tissue. To verify that the OCT-4 positive cells were fibroblasts, we co-stained tissues for the fibroblast marker vimentin and OCT-4 (Fig. 4). Co-staining indicated that OCT-4 localized in the vicinity of vimentin positive cells, with several instances of co-localization (yellow, Fig. 4), suggesting that these cells are indeed fibroblasts. Our data suggest the presence of three populations of cells: OCT-4^+^, vimentin^+^, and a subset of cells positive for both OCT-4 and vimentin. MDA-MB-231 is a human breast cell carcinoma cell line that has high intrinsic expression of OCT-4. We did not evaluate the subcellular distribution of OCT-4 or vimentin in the tissue sections.

Literature values and independent pH measurements in our laboratory show that the pH in the tumor area ranges from 6.5–7.0. By chronically maintaining the extracellular pH below 7, cancer cells can stimulate non-tumor cells to reprogram via pH-related stress. This process could aid the recruitment of surrounding cells into the tumor matrix.

## Discussion

The extracellular pH of tumors has been implicated in the metastasis and behavior of tumor cells, and changes in this pH could modify the downstream behavior of cancer^18^. To our knowledge, this is the first report of the expression of OCT-4 in the vicinity of tumors, particularly in supporting stromal cells. Stress based *in vitro* OCT-4 expression has been reported previously for stem cells^14^,^21^. Our data suggests that chronic mild acidity, which is present in the tumor environment along with other stressors, is capable of inducing OCT-4 expression in murine fibroblasts and may be sufficient to induce OCT-4 expression *in vivo*. These observations suggest that tumor cells *in vivo* may be capable of reprogramming surrounding stromal cells using environmentally mediated factors.

There are a few caveats to the above platform. The tumors were all xenografts and it is not known if this phenomenon occurs in spontaneous tumor models. We focused primarily on murine fibroblasts to facilitate *in vivo* assessment of the phenomenon with the same cells. It is also unclear if the *in vivo* OCT 4 expression changes with tumor size in this study.

*Ex vivo* histology showed expression of OCT-4 in cells that are not fibroblasts. Other than MDA MB-231 tumors, which had high intrinsic expression of OCT-4, the positive cells did not make up the bulk of the tumor mass. We posit that these cells may be endothelial or other epithelial cells in the stromal region, or perhaps some small subset of cancer cells. This hypothesis is supported by *in vitro* expression of OCT4^+^ in PMEC cells, a primary mammary epithelial cell line, under acidic conditions.

This report offers observational evidence that mild acidity does induce some degree of OCT-4 expression *in vivo*, therefore confirming previous findings about stress based OCT-4 expression. However, it is not clear how long these cells maintain the OCT-4 expression nor if the expression induces any other pluripotent stem cell phenotype. To rule out the possibility of artifact, we tested both a mouse monoclonal and a rabbit polyclonal anti-OCT-4 antibody. We observed similar results with both antibodies, although OCT-4 localization at times alternated between cytoplasmic (murine monoclonal) and nuclear (rabbit polyclonal), depending on the type of antibody used (Fig. 1, Supplementary Figure 1).

Fibroblasts are traditionally considered to be strong supporting actors in the tumor microenvironment. The concept that chronic physiological extracellular acidity can have significant effects on the expression of reprogramming markers, such as OCT-4 both *in vitro* and *in vivo*, suggests a possible mechanism for the phenotypic changes in cancer associated fibroblasts. One report indicates that acidity may actually transform fibroblasts into cancerous tissue^22^. Some preliminary data (Supplementary Figure 4) demonstrate that implantation of these acid treated cells induces tumor growth in athymic nude mice. For this study, fibroblast cells were not co-cultured with tumor cells. Further, the concept that acid mediated stress can induce the presence of factors that cause reprogramming *in vivo* raises the question of what happens if intratumoral pH is changed *in vivo*. Indeed, pH changes *in vivo* can inhibit tumor growth^23^.

Overall, this study reveals that the stroma of tumors express OCT-4. Based on *in vitro* data, it is likely that chronic mild acidic stress could facilitate cell reprogramming. Given the ubiquity of extracellular acidic environments in some solid cancers, this process could be a common occurrence *in vivo*. We demonstrated that pH evidently has an important effect on both cancer cells and the supporting stroma. These results further support the relevance of the extracellular matrix and its environment to cancer behavior, prognosis, and therapy. In particular, the increasing evidence that tumors actively maintain an extracellular acidic environment implies a causative role for pH in tumor survival and proliferation. There is thus an intersection between the significant work involved in cancer metabolomics, the Warburg effect, and the tumor microenvironment. Future work will focus on replicating this observed phenomenon *in vivo* in human subjects.

## Methods

### Immunofluorescent staining

Cells or tissues were stained per the manufacturer’s protocol using rabbit polyclonal (ab19857, Abcam, Cambridge, MA; 1:200 dilution) or murine anti-OCT-4 (ab91194, Abcam, Cambridge, MA; 1:200 dilution), and rat anti-Vimentin (ab115189, Abcam, Cambridge, MA; 1:200 dilution). Secondary antibodies were Allophycocyanin anti-mouse IgG (A-865, Life Technologies, Grand Island, NY; 1:1000), AlexaFluor 555 anti-rabbit IgG, and anti-Rat IgG AlexaFluor 647 (Abcam, Cambridge, MA; 1:1000 dilution). Cells were visualized with an Olympus FV1000 Confocal Microscope using 488 nm, 543 nm, or 633 nm for excitation. Tissue was visualized with an Olympus BX51 upright epifluorescence microscope (Olympus America, Center Valley, PA) using excitation/emission filters of 480 nm/535 nm, and 620 nm/700 nm.

### Tumor model growth

All the animal experiments were conducted in accordance with the approved guidelines for the care and use of laboratory animals in research, and the protocol was approved by the Washington University Animal Welfare Committee. BxPC-3, MDA-MB-231, and EMT-6 cells were injected subcutaneously into the dorsal flanks of athymic nude mice, while 4T1luc cells were injected into Balb/c mice. Mice were sacrificed when the tumors were between 0.5 and 1.5 cm in diameter. In certain cases, tumors may have had exposure to the near-infrared tumor imaging agent cypate prior to excision. Tumors were excised based on gross appearance and frozen in OCT media. The average pH in 4T1 tumor models was 6.8+/− 0.1 pH units, as measured by an invasive probe (5 mm probe). The average pH level in EMT-6 and BxPC-3 tumors was not measured, previous studies reported acidity in BxPC-3 and EMT-6 tumor models and the importance of the pH for chemotherapy resistance^19^. Tissue sections were cryosectioned at 10 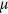
m thickness. Tissue was visualized with an Olympus BX51 upright epifluorescence microscope (Olympus America, Center Valley, PA) using excitation/emission filters of 480 nm/535 nm, and 620 nm/700 nm.

### Cell culture

MDA-MB 231, 4T1luc, and BxPC-3 tumor cells, and 3T3-GFP fibroblasts were cultured alone or in co-culture in acidic (pH 6.5), slightly acidic (pH 6.8), or non-acidic (pH 7.4) media (DMEM + 10% FBS + 1% Pen Strep) in 8 well slides. Acidic media was created by titrating DMEM + 10% FBS + 1% Pen Strep media with 1 M HCl to the desired pH range. Slides were incubated for 7 days without any modifications in 5% CO2 at 37 °C, after which they were stained for OCT-4.

### Longitudinal study

Cells were cultured as described above in normal media overnight and then switched to acidic conditions at day 0, 1, 3, and 5 and incubated until day 7 prior to immunostaining.

### 3T3 Fibroblast growth in nude mice

3T3-GFP Fibroblast cells were grown in T75 flasks under either HCl titrated, pH 6.5 conditions, as described above, or in pH 7.4 media for 7 days, or grown in T75 flasks under normal conditions overnight. Cells were trypsinized and injected in a 50% matrigel/PBS matrix. For each “tumor” injection site, 1 million cells from the above three categories were used. Mouse 1 received the acid treated cells on its left flank and 7 day pH 7.4 treated cells in its right flank. Mouse 2 received the acid treated cells on its left flank and 1 day pH 7.4 treated cells in its right flank. Mouse 3 received 7 day control cells on its left flank and 1 day pH 7.4 treated cells in its right flank. GFP images were taken 2 weeks later using the ART Optix MX3 system.

## Additional Information

**How to cite this article**: Som, A. *et al*. Acidic extracellular pH of tumors induces octamer-binding transcription factor 4 expression in murine fibroblasts *in vitro* and *in vivo*. *Sci. Rep.* **6**, 27803; doi: 10.1038/srep27803 (2016).

## Supplementary Material

Supplementary Information
