# Supplementary material for: Acidic extracellular pH of tumors induces octamer-binding transcription factor 4 expression in murine fibroblasts in vitro and in vivo
Source: Sci Rep. 2016 Jun 15;6:27803. doi: 10.1038/srep27803 (PMC4908587; doi:10.1038/srep27803)
Supplement: Supplementary Information [file srep27803-s1.pdf]

Acidic extracellular pH of tumors induces octamer-binding transcription factor expression 4 in murine fibroblasts *in vitro* and *in vivo*

Avik Som[1,2,] Sharon Bloch[1], Joseph E. Ippolito[1],[2], Samuel Achilefu[1,2,*]

[1] Department of Radiology, Washington University in St. Louis School of Medicine, St. Louis, Missouri, 63110, USA
[2] Department of Biomedical Engineering, Washington University in St. Louis School of Medicine, St. Louis, Missouri, 63110, USA

Supplementary Figures

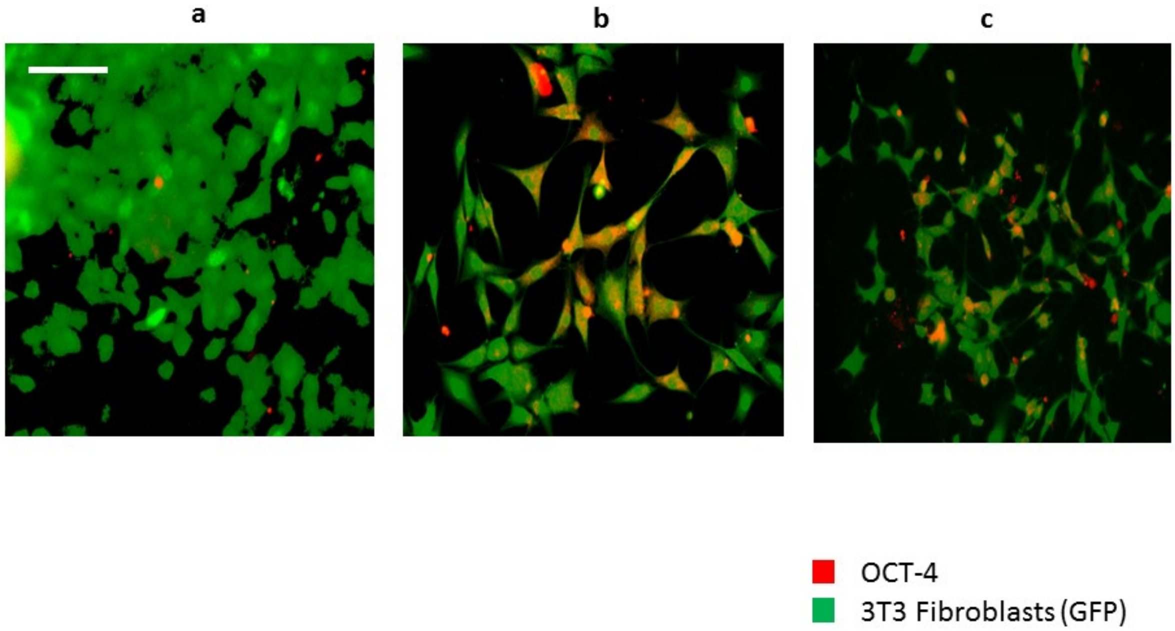

Supplementary Figure 1: Murine monoclonal anti-OCT 4 staining in 3T3 fibroblast cells under different conditions. (a) OCT-4 staining of GFP^+ fibroblasts cultured in normal media for 7 days. **(b)** OCT-4 staining of GFP^+ fibroblasts cultured in acidified media (pH 6.5) for 7 days. **(c)** OCT-4 staining of GFP^+ fibroblasts co-cultured with MDA-MB-231 tumor cells in acidified media (pH 6.5) for 7 days. Red represents OCT-4^+ cells, and Green represents GFP+ fibroblasts. Scale bar represents 100 μm and is at 40x magnification. Note Supplementary Figure 1b is a replicate of Figure 2f in the main text and is used here for comparison.

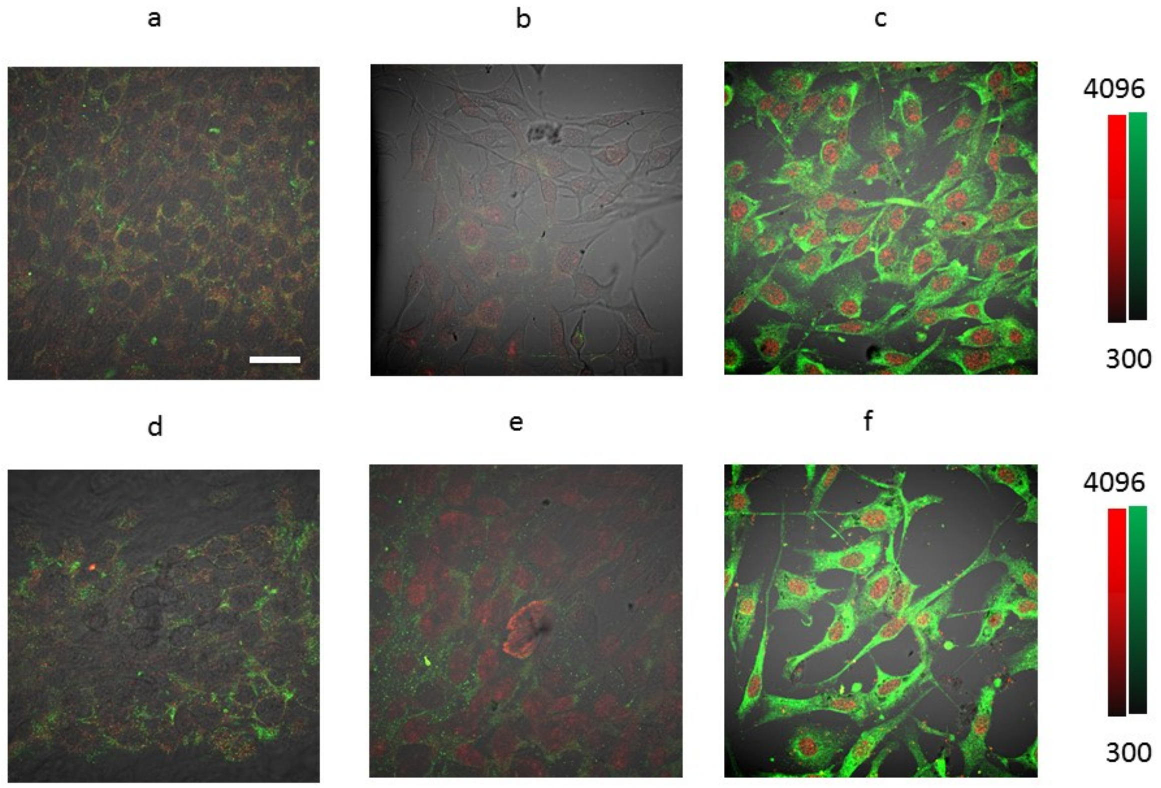

Supplementary Figure 2: Effect of pH gradient on OCT-4 expression in 3T3 fibroblast cells. **(a)** Fibroblasts incubated in media (pH 7.4) for 7 days without changing the media. **(b)** Fibroblasts incubated in acidified media (pH 6.8) for 7 days without changing the media. **(c)** Fibroblasts incubated in acidified media (pH 6.5) for 7 days without changing the media. **(d)** Fibroblasts co-cultured with MDA-MB-231 breast cancer cells incubated in acidified media (pH 7.4) for 7 days. **(e)** Fibroblasts co-cultured with MDA-MB-231 breast cancer cells incubated in acidified media (pH 6.8) for 7 days. **(f)** Fibroblasts co-cultured with MDA-MB-231 breast cancer cells incubated in acidified media (pH 6.5) for 7 days. Scale bar is 100 µm. Red represents OCT-4^+ cells, and Green represents Vimentin$^+$ fibroblasts.

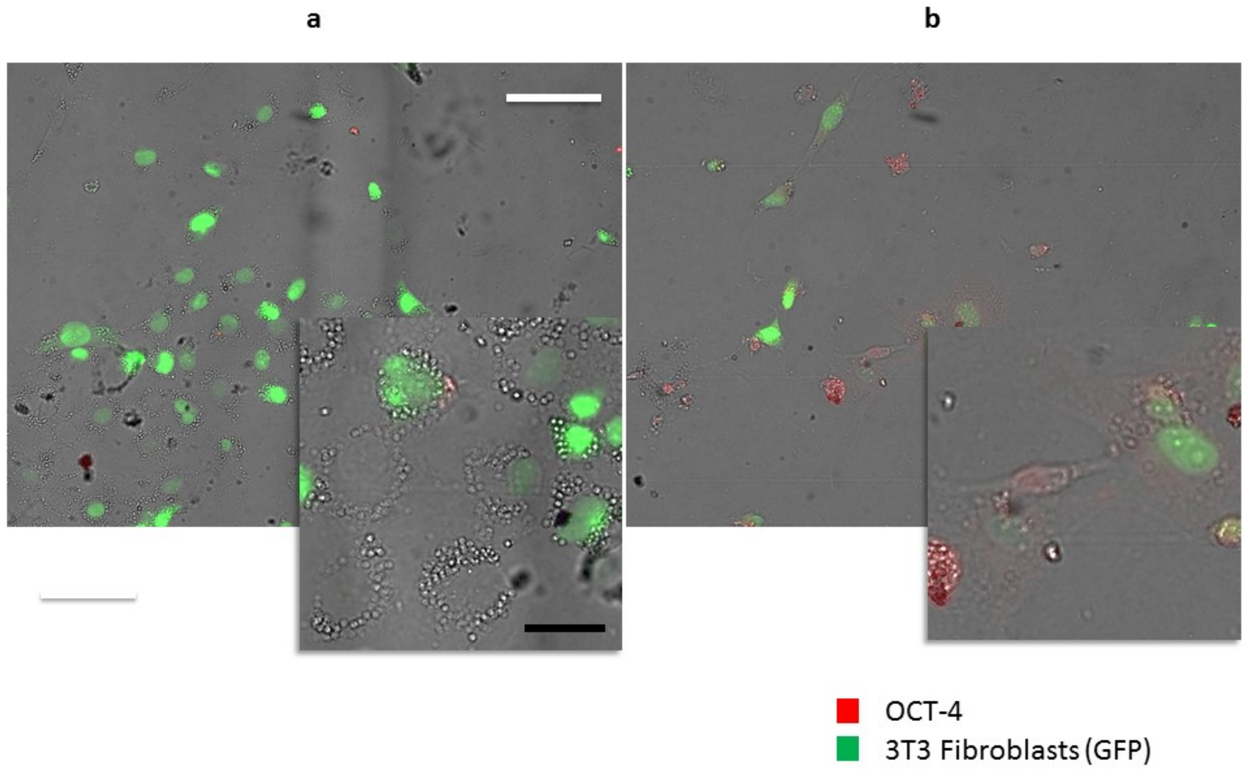

Supplementary Figure 3: Co-Culture of Fibroblast-GFP cells with primary mammary epithelial (PMEC) cells. (a) Co-culture of PMEC cells with 3T3-GFP-fibroblasts for 7 days, and stained with OCT-4. **(b)** Co-culture of PMEC cells with 3T3-GFP-fibroblasts in acidified media for 7 days. Cells without GFP are primary mammary epithelial cells.

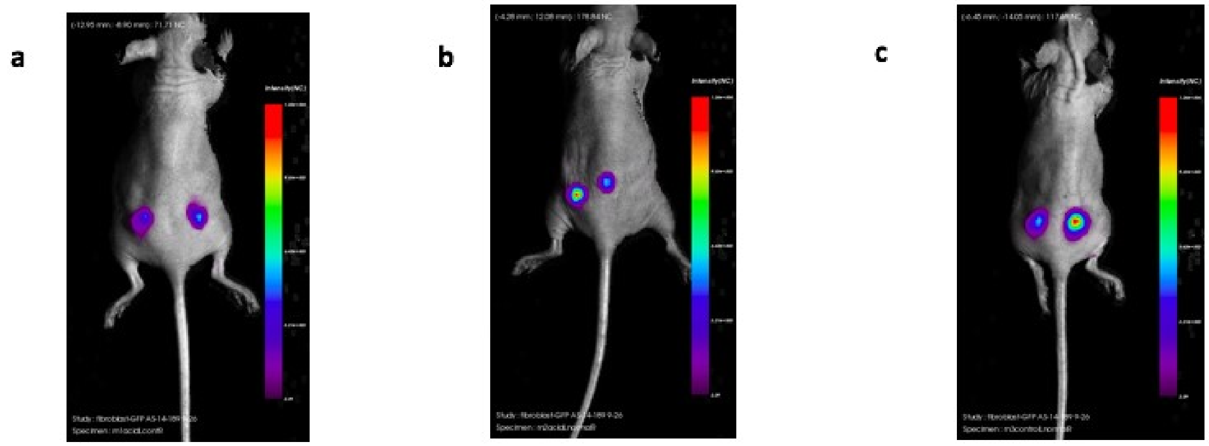

Supplementary Figure 4: Dorsal GFP images showing in vivo growth of acid treated fibroblasts. (a) GFP image of mouse 1: acid treated and 7 days pH 7.4 treated 3T3 cells on the left and right flanks respectively. **(b)** GFP image of mouse 2: acid treated and 1 day pH 7.4 treated 3T3 cells on the left and right flanks, respectively. **(c)** GFP image of mouse 3: 7 days control and 1 day pH 7.4 treated 3T3 cells on the left and right flanks, respectively.

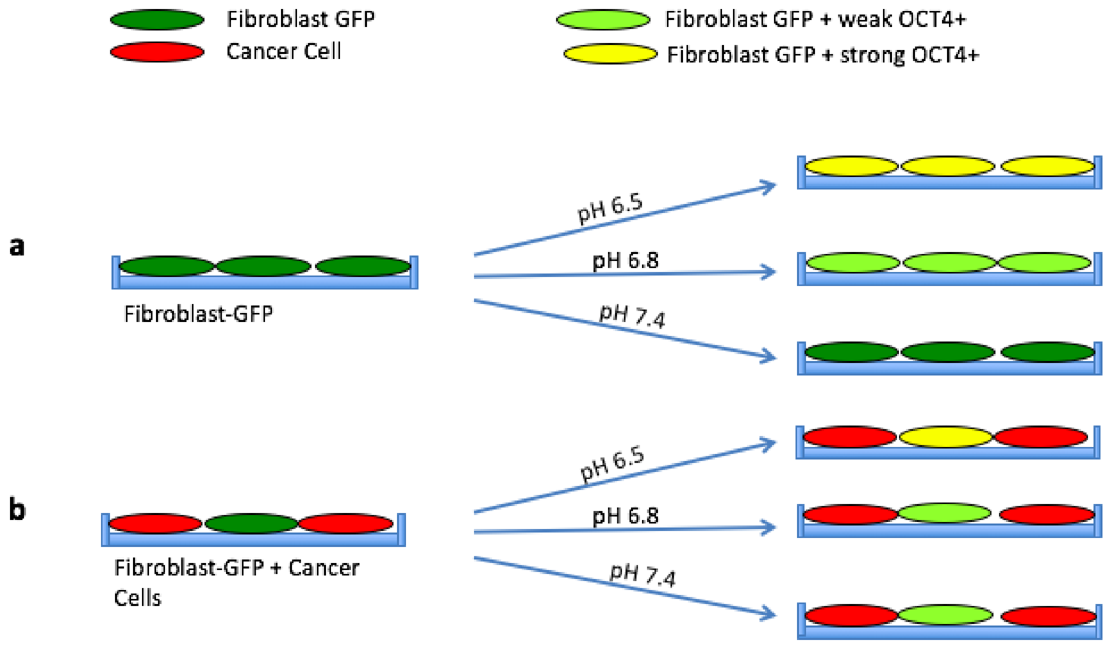

Supplementary Figure 5: Schematic for OCT-4 induction *in vitro.* (a) Treatment of 3T3-GFP^{+} with pH 6.5 medium induces the strongest OCT-4 expression, with decreasing expression towards 7.4. **(b)** Co-culture of 3T3-GFP^{+} with a tumor cell line can induce similar effects with the strongest OCT-4 expression at pH 6.5.

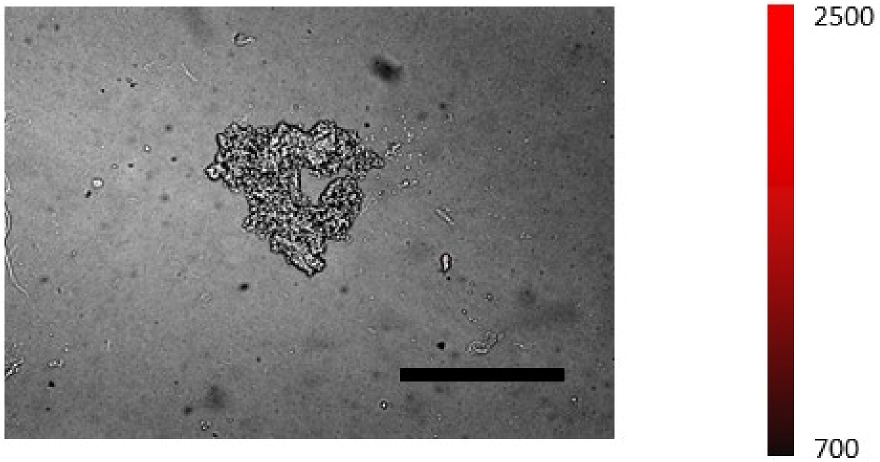

Supplementary Figure 6: Negative control for anti-OCT-4 using heart muscle stained for anti-OCT-4.
